# A significant therapeutic effect of silymarin administered alone, or in combination with chemotherapy, in experimental pulmonary tuberculosis caused by drug-sensitive or drug-resistant strains: *In vitro* and *in vivo* studies

**DOI:** 10.1371/journal.pone.0217457

**Published:** 2019-05-30

**Authors:** Edén M. Rodríguez-Flores, Dulce Mata-Espinosa, Jorge Barrios-Payan, Brenda Marquina-Castillo, Mauricio Castañón-Arreola, Rogelio Hernández-Pando

**Affiliations:** 1 Department of Pathology, Experimental Pathology Section, National Institute of Medical Sciences and Nutrition ‘‘Salvador Zubiran”, Mexico City, Mexico; 2 Genomic Sciences Program, Autonomous University of México City, Mexico City, México; Rutgers Biomedical and Health Sciences, UNITED STATES

## Abstract

For many years, tuberculosis (TB) has been a major public health problem worldwide. Advances for treatment and eradication have been very limited. Silymarin (Sm) is a natural product with antioxidant and hepatoprotective activities that has been proposed as a complementary medicine to reduce the liver injury produced by the conventional anti-TB chemotherapy. Sm also has immunoregulatory and microbicide properties. In this study, we determined the effect of Sm on the growth control of mycobacteria. *In vitro* studies showed that Sm and Silibinin (the principal active compound of Sm) have microbicidal activity against drug-sensitive and multidrug-resistant (MDR) mycobacteria, induce the production of protective cytokines from infected macrophages, and improve the growth control of mycobacteria (p ≤ 0.0001). Studies *in vivo* using a model of progressive pulmonary TB in BALB/c mice infected with drug-sensitive or MDR mycobacteria have shown that Sm induces significant expression of Th-1 cytokines such as IFN-γ and IL-12 as well as TNFα, which produce significant therapeutic activity when administered alone and apparently have a synergistic effect with chemotherapy. These results suggest that Sm has a bactericidal effect and can contribute to the control and establishment of a T_H_1 protective immune response against mycobacterial infection. Thus, it seems that this flavonoid has a promising potential as adjuvant therapy in the treatment of TB.

## Introduction

Tuberculosis (TB) is an ancient infection caused by *Mycobacterium tuberculosis*. According to a WHO report, in the past year, 10.4 million people have developed active TB worldwide, and 1.4 million die each year [1, 2, 3]. TB is now the leading cause of death worldwide caused by an infectious agent—surpassing malaria and HIV [4]. Anti-TB treatment has been available for more than 60 years but requires the administration of four antibiotics for at least six months, confers a risk of developing side effects (including gastric, neurologic and hematological alterations) and is potentially hepatotoxic [5,6,7]. The long treatment duration, coupled with adverse effects, affects patient adherence to treatment, decreasing its efficiency and resulting in the emergence of drug-resistant TB [7, 8, 9, 10, 11, 12]. In recent years, the world has seen a rapidly emerging epidemic of multidrug-resistant (MDR) TB, and extensively drug-resistant (XDR) TB, which is frequently lethal and extremely expensive and complicated to treat [13, 14].

In the last several years, plants and their derivatives have been used as an alternative in the treatment of diverse diseases [15]. To reduce the hepatic damage produced by the treatment, some authors have proposed the use of natural complementary therapies and even the use of herbal extracts with hepatoprotective activity [16, 17, 18, 19]. In countries such as China, hepatoprotective drugs are prescribed alongside anti-TB treatment [20, 21, 22]. One of the most used treatments for liver disorders is a standardized extract of silymarin (Sm), which is obtained from the seeds of the milk thistle *Silybum marianum* and is mainly composed of silibinin (Sb) (60–70%), silydianin and silychristin [23]

Sm has been used for centuries as a natural supplement for liver illness or as a treatment in cases of chronic liver diseases such as cirrhosis [24, 25]. In addition to its hepatoprotective effect [24,26], Sm also has other activities, such as antioxidant, immunostimulatory and anti-inflammatory activities, which improve the outcomes in hepatic diseases and diminishes hepatic inflammation and fibrosis [27]. Recent studies have demonstrated that Sm also has antibacterial activity against gram-positive bacteria, such as *Bacillus subtilis* and *Staphylococcus epidermidis* [28]. Sm also shows significant synergistic activity in modulating the effect of aminoglycosides [15, 29]; and displays some anti-inflammatory and immunoregulatory activities [30,31]. Sm has an inhibitory action in the production of proinflammatory mediators produced by lymphocytes [32], and in the release of histamine by basophils [33]. In macrophages, it seems that Sm modulates the activation of the nuclear factor-kappa B (NF-κB) pathway [11, 34]; and the expression of inducible nitric oxide synthase [35, 36]. Additionally, Sm has demonstrated immunomodulatory function in dendritic cells [37]. Most of the pharmacological properties are attributed to Sb, which is the main constituent of Sm [38]. Sb can favor the T_H_2 immune response in dendritic cells and reduce chronic inflammation in a dose-dependent manner [39]. All these activities could interfere with the protective immune response during TB or favor the activation of latent infection [40].

Recently, it was demonstrated that the coadministration of Sm significantly reduces the biochemical alterations and histological damage caused by anti-TB treatment in an animal model [41, 42]. However, the hepatoprotective activity in TB patient remains controversial [22,43]. Although Sm has been used as a natural medicine for more than 2000 years [44], the effects of its immunomodulatory and anti-inflammatory activities during mycobacterial infections remain unknown. However, the Sm properties of safety, easy availability and low cost make it a promising treatment of natural origin. In this work, we investigated the direct effect of Sm on mycobacteria viability, its effect in human monocyte-derived macrophages infected with mycobacteria and the therapeutic effect in infected mice with drug-sensitive and MDR strains, treated with Sm alone or in combination with chemotherapy.

## Material and methods

### *Mycobacterium tuberculosis* strains

Evaluation of *in vitro* and *in vivo* anti-TB activity was carried out, using the reference strain of *M*. *tuberculosis* H37Rv ATTC 27294, which is susceptible to all five first-line anti-TB drugs, and the clinical isolate CIBIN 99 (MDR), which was previously characterized and is resistant to streptomycin, isoniazid (INH), rifampicin (RIF), ethambutol (EMB) and pyrazinamide (PZA) [45].

### Sm and Sb preparation for *in vitro* experiments

Sm and Sb (Sigma-Aldrich, USA) were solubilized in DMSO and prepared as a 100 mM stock solution. For each experiment, a fresh working solution was prepared by diluting the stock solution with serum-free RPMI-1640 media (Sigma-Aldrich, USA).

### Monocyte-derived macrophages

Buffy coats from healthy donors were collected in the Blood Center of the Centro Médico Nacional 20 de Noviembre or in the Blood Center of the Instituto Nacional de Ciencias Médicas y Nutrición Mexico, under the approval of the Institutional Ethics Committee. Human monocyte-derived macrophages (hMDMs) were purified from peripheral blood mononuclear cells (PBMCs) from freshly collected blood samples. PBMCs were isolated by density gradient centrifugation on Histopaque-1077 (Sigma-Aldrich, USA) according to the manufacturer’s recommendations. PBMCs were washed with PBS 1X and cultured in RPMI-1640 medium supplemented with 10% (v/v) of heat-inactivated fetal bovine serum (FBS; Gibco BRL, Grand Island, NY) for 2 h at 37°C in a humidified atmosphere with 5% CO_2_ to allow monocytes to adhere to the plastic plate. Non-adherent cells were eliminated by washing, and adherent cells, enriched for monocytes, were cultured for 7 days for macrophage differentiation in RPMI-1640 medium supplemented with 10% FBS at 37°C in a humidified atmosphere with 5% CO_2_. Cells were seeded at either at 6×10^4^, 5×10^5^ or 1.2×10^6^ cells per well in 96, 12 or 6-well tissue culture plates, respectively. Differentiation was confirmed by determining the CD14 expression by flow cytometry using an anti-CD14-FITC antibody (BD Biosciences).

#### Ethics statement

The Ethics Committee of the “Centro Médico Nacional 20 de Noviembre” approved the project: “Validation of deregulated miRNAs in macrophages derived from monocytes after stimulation with *M*. *tuberculosis* and *M*.*bovis* proteins” by Dr. Sofia Lizeth Alcaraz Estrada. The cells used in this study were used for this project.

### Cytotoxicity determined by a neutral red assay

For cytotoxicity determinations, we used a neutral red-based *in vitro* toxicology assay kit (Sigma-Aldrich, USA), according to the manufacturer’s recommendations. Macrophages (Mø) were stimulated with 50, 100, 150, 200 or 250 μM of Sm or Sb. After 3, 6, 12, 24 and 48 h of incubation, the cytotoxicity was evaluated. Cells treated with a concentration of DMSO equivalent to that used in the high-concentration flavonolignan test wells were used as controls. Three hours before each evaluation time, the medium was replaced with medium containing 10% [v/v] neutral red solution and incubated for 3 h to allow dye uptake. Then, Mø were washed with PBS containing 0.5% [w/v] formaldehyde + 1% [w/v] calcium chloride, and the dye was extracted from the intact viable cells with a solution of 1% [v/v] acetic acid and 50% [v/v] ethanol. The plate was incubated for 10–15 minutes at room temperature and then mixed on a microplate shaker for 10 minutes. Finally, 100 μl samples were transferred to a 96-well plate, and the absorbance was measured at 540 nm. The viability index was calculated as: (OD treated cells / OD untreated cells).

### Cytokine expression analysis

To analyze cytokine expression in hMDMs, cells were stimulated for 24 h with Sm or Sb at 50 and 100 μM doses. Brefeldin A (10 μg/ml; Sigma) was added to the cultures for the last 4 h of stimulation. Stimulated hMDMs were washed twice with PBS 1X, trypsinized, and resuspended on staining buffer (PBS 1X, 1% heat-inactivated FCS, 0.09% sodium azide, pH 7.4). Fc receptors were blocked by incubating the cells with 10% normal human serum in staining buffer for 15 minutes at 4°C. Then, Mø were washed twice with staining buffer, resuspended in a permeabilization solution (PBS 1X with 1% heat-inactivated FCS, 0.1% sodium azide, 0.1% saponin, pH 7.4) and incubated for 20 minutes at 4°C; washed two times with washing buffer (PBS 1X with 0.09% sodium azide, 1% Saponin, pH 7.4); and then thoroughly resuspended in permeabilization buffer. Immunolabeling was performed with IFN-γ-PE, IL-12-FITC; NF-κβ-PE or TNF-α FITC antibodies (all from BD Pharmingen, USA) incubated for 15 min at room temperature. Then, cells were incubated in the dark for 30 min at 4°C, washed two times with washing buffer, resuspended in FACSFlow (BD Biosciences, USA) and analyzed on a FACSCalibur flow cytometer. The results are expressed as the percentage of positive cells for each cytokine.

### Minimum inhibitory concentration (MIC) determination

Briefly, bacterial cells were plated to achieve a final inoculum of 1.5 x 10^5^ cells per well in a 96-well cell culture treated dish. All wells contained 100 μL of 7H9-OADC supplemented growth media. One hundred microliters of the diluted flavonoids at the highest concentration starting at 800 μM (482 μg/ml) was added to one well, the contents of these wells were mixed thoroughly, and 100 μL was transferred into the next well; the process was then repeated, thus creating serial two fold-dilutions ranging from 6 to 800 μM (3.123–482 μg/ml). A row containing only DMSO was also tested to show the antibacterial effect of the solvent (not shown). In addition, INH (μg/ml) was used as a control, and medium without any compound was used as a negative control. Plates were incubated at 37°C with 5% CO2 for 7 days in a humidified incubator. After 5 days of incubation, the MIC was determined as the lowest concentration of the agent that completely inhibited visible growth. Changes in proliferation induced by Sm treatment were measured with a Cell Titer 96 Aqueous One Solution Cell Proliferation assay reagent (Promega, Madison, WI) according to the manufacturer’s instructions. Four hours prior to the end of each exposure period, an MTS mixture (20 μL/well) was added. MIC values were determined spectrophotometrically at 492 nm (BioTek Instruments, ELX 800, USA). The antimicrobial activity was confirmed by subculturing MIC tests to fresh agar plates. Four experiments with three replicates per treatment were performed. The results were analyzed using a 2-way ANOVA test with Sidak’s comparison test.

### Determination of the synergistic effect of silymarin and antitubercular drugs

The possible synergy of Sm with anti-TB drugs was determined by the MIC method as described above. We used the reported MIC (1X) for first- and second-line drugs and half (0.5X) of the reported dose [10], using each drug alone or in combination with Sm or Sb. For first-line antibiotics, the 1X doses were as follows: INH (0.1 μg/ml), RIF (0.1 μg/ml), and PZA (5.6 μg/ml). For second-line antibiotics, the following doses were tested: ethionamide (5.6 μg/ml), amikacin (4 μg/ml) and moxifloxacin (4 μg/ml). The concentration used for Sm and Sb was 30 μg/ml (50 μM).

### CFU determination in infected macrophages

To determine the bacillary loads in Mø treated with Sm or Sb, hMDM cells were cocultured for 4 h with *M*. *tuberculosis* H37Rv or clinical isolate CIBIN 99 (MDR) strains at a multiplicity of infection (MOI) of 5:1. Next, the cells were washed 3 times with fresh RPMI 1640 medium to remove unphagocytosed bacteria. The cells were subsequently treated with 50 and 100 μM of Sm or Sb and were incubated for 24 h. Mø were lysed with lysis solution (0.1 M Tris, pH 7.6 containing 0.05% [w/v] SDS in H_2_O), and the CFUs were determined by plating 10-fold serial dilutions onto Middlebrook 7H10 agar media supplemented with OADC. CFUs were counted after 2–3 weeks of incubation at 37°C in 5% CO_2_. The results are expressed as the mean of three independent experiments. Statistical analysis was performed using one-way analysis of variance (ANOVA) for multiple observations.

### Experimental model of progressive pulmonary tuberculosis in BALB/c mice

The experimental model of progressive pulmonary TB has been previously described in detail [46]. Briefly, specific pathogen-free male BALB/c mice, 6–8 weeks of age, were anesthetized (Sevoflurane; Abbott Laboratories, IL, USA) and infected with *M*. *tuberculosis* strain H37Rv or CIBIN99 by the endotracheal route (i.t.) with the administration of 2.5x10^5^ viable bacteria suspended in 100 μL of PBS. Infected mice were maintained in groups of five in cages fitted with microisolators connected to negative pressure. All procedures were performed in a biological security cabinet at a biosafety level III facility.

### Animal care and housing

All animal work was carried out according to the guidelines and approval of the Ethical Committee for Experimentation in Animals of the National Institute of Medical Sciences and Nutrition (INCMNSZ) in Mexico City, permit number CINVA 1825 PAT-1825-16/18-1. Mice were maintained in individually ventilated cages and adverse clinical signs, such as weight loss, hunched posture, dehydration, rough hair coat, etc., were monitored to determine humane endpoints.

### Sm *in vivo* administration

Sm was solubilized into a nanoemulsion formulation containing 35% w/w of mix (mixture of Tween 80 as a surfactant and ethanol as a cosurfactant in the ratio of 2:1) and double distilled water as the aqueous phase (65% w/w) to increase bioavailability [47]. To evaluate the effect of Sm, animals surviving 60 days after infection with the drug-sensitive strain H37Rv or the MDR strain were randomly allocated into four treatment groups: 1) animals treated every day with 5 mg of Sm administered by the intragastric route (i.g.); 2) animals infected with the drug-sensitive strain and treated daily (i.g.) with the first-line antibiotics 10 mg/kg RIF, 10 mg/kg INH, and 30 mg/kg pyrazinamide dissolved in isotonic saline solution, as well as mice infected with the MDR strain and treated with 3 mg/kg of moxifloxacin, 6 mg/kg of ethionamide and 7.5 mg/kg of PZA; 3) animals treated with both antibiotic and Sm and 4) infected mice that only received vehicle as a control group under the same procedure. Groups of six animals were euthanized by exsanguination under terminal anesthesia after 7, 14, 30 and 60 days of treatment. Two independent experiments were carried to validate results.

### Assessment of colony-forming units (CFU) in infected lungs

Immediately after the animals were euthanized by exsanguination, the lungs were removed and immediately frozen by immersion in liquid nitrogen. For CFU determination, frozen lungs were disrupted using ceramic beads in tubes with 1 ml of PBS containing 0.05% Tween. Four dilutions of each homogenate were spread onto duplicate plates containing Bacto Middlebrook 7H10 agar (Difco BD, Sparks, MD, USA) enriched with OADC (Difco). The colonies was counted after 15 and 21 days of incubation at 37°C with 5% CO_2_

### Preparation of tissue for histology and morphometry

Parasagittal sections were dehydrated and embedded in paraffin, sectioned at 5-μm width, and stained with hematoxylin and eosin (H&E). The percentage of lung area affected by pneumonia was measured using a Leica Q-win Image Analysis System (Cambridge, UK). Measurements were performed in a blind manner, and data are expressed as the mean of four animals ± the standard deviation (SD).

### Real-time PCR expression analysis of cytokines in infected lungs

Lung lobes were homogenized in RLT buffer and used to isolate total RNA, using an RNeasy Mini Kit (QIAGEN Mexico, Colima, Mexico) according to the recommendations of the manufacturer. The quality and quantity of RNA were evaluated with spectrophotometry (260/280 ratio, NanoDrop1000; Thermo Fisher Scientific, Waltham, MA, USA) and electrophoresis on agarose gels. Reverse transcription of the mRNA was performed using 100 ng of RNA and an Omniscript kit with oligo-dT (QIAGEN Mexico). Real-time PCR was performed using a 7500 real-time PCR system (Applied Biosystems, Foster City, CA, USA) and a QuantiTect SYBR Green Master Mix kit (Qiagen). Standard curves of quantified and diluted PCR product, as well as negative controls, were included in each PCR run. Specific primers were designed for the following targets: acidic ribosomal protein (RLP0) as a housekeeping gene: 5′-CTCTCGCTTTCTGGAGGGTG-3′, 5′-ACGCGCTTGTACCCATTGAT-3; interleukin 12 (IL-12): 5′-CAGAAGCTAACCATCTCCTGGTTTG-3′, 5′-CCGGAGTAATTTGGTGCTCCACAC-3; tumor necrosis factor alpha (TNF-α): 5′-TCGAGTGACAAGCCTGTAGCC-3′, 5′-TTGAGATCCATGCCGTTGG-3′, and interferon gamma (IFN-γ): 5′-GGTGACATGAAAATCCTGCAG-3′, 5′-CCTCAAACTTGGCAATACTCATGA-3′. The cycling conditions used were as follows: initial denaturation at 95 °C for 15 min, followed by 40 cycles at 95 °C for 20 s, 60 °C for 20 s, and 72 °C for 34 s. The quantities of the specific mRNA in the sample were measured according to the corresponding gene-specific standard. The mRNA copy number of each cytokine was related to 1 million copies of mRNA encoding the RPLP0 housekeeping gene.

### Tuberculosis reactivation trial after treatment with silymarin

After two months of infection, a group of mice was treated for two months with silymarin, as mentioned above. At the end of the treatment, the mice were kept without any treatment for one month. Subsequently, the mice were treated with corticosterone (3 mg/L) dissolved in drinking water to induce reactivation. After 1 month of corticosterone supplementation, the mice were sacrificed, and the effect of the treatment was evaluated by counting the colony forming units (CFU) in lung homogenates.

### Statistical analysis

Values are presented as the means ± SEM of three independent experiments. The data were analyzed by parametric two-way ANOVA with Tukey’s posttest or a nonparametric Kruskal Wallis multiple comparison test. Analyses were performed using GraphPad Prism version 6 for Mac OS X, GraphPad Software, La Jolla, California USA. For all analyses, a P value <0.05 was considered statistically significant.

## Results

### *In vitro* studies

**Fig 1:**
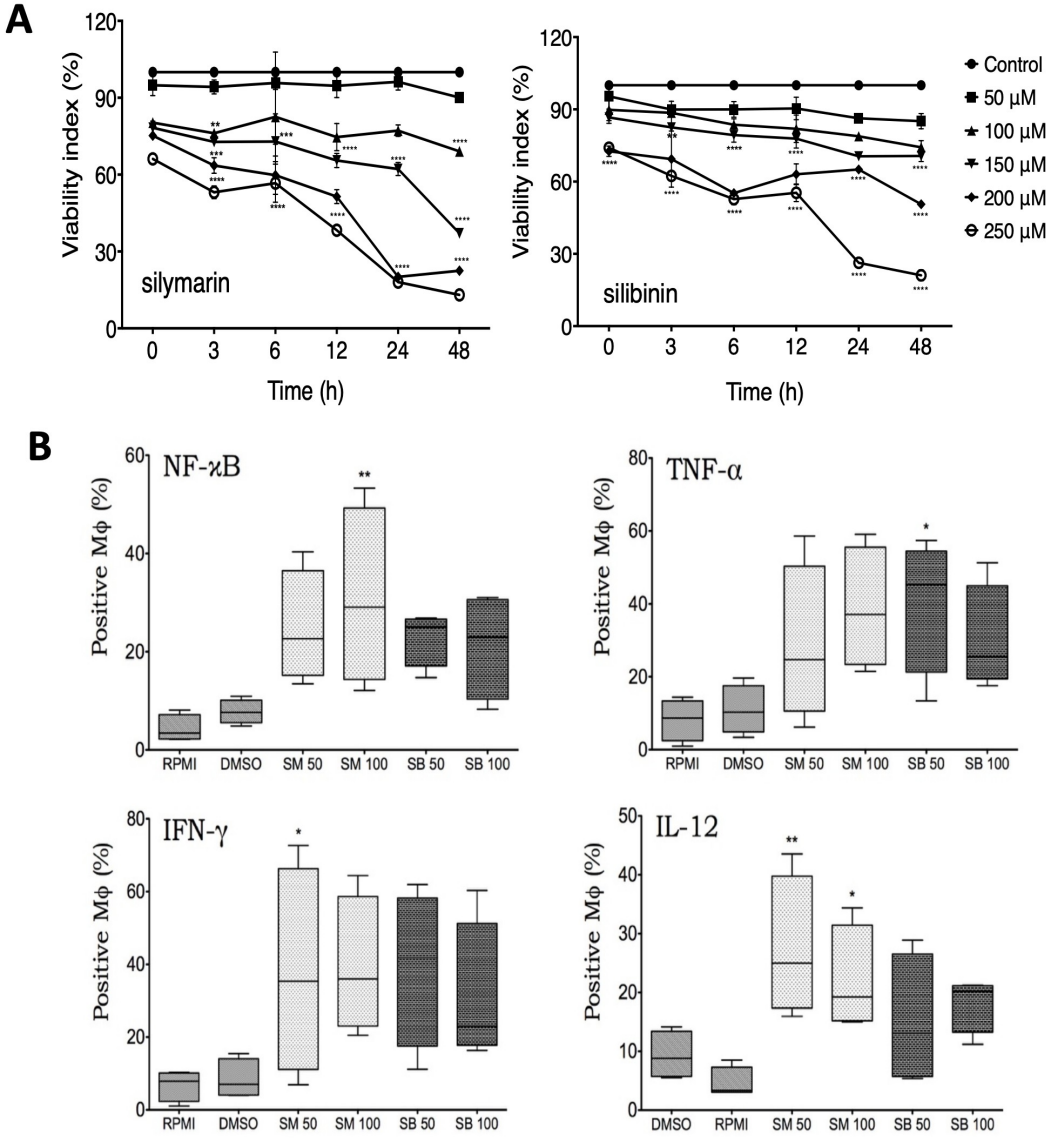
Effects of silymarin and silibinin on cell viability and production of cytokines in noninfected human macrophages. A) hMDMs were stimulated with the indicated concentrations of silymarin (Sm) or silibinin (Sb), and the cell viability was determined at different time points using the neutral red assay. A dose-time-dependent cytotoxicity effect of treatment was more evident in Mø stimulated with Sm than in those stimulated with Sb. The data represent the mean ± SEM of individual determinations of four independent experiments performed in duplicate. * *p* ≤ 0.05. B) After incubation during 24 h with Sm or Sb, the percentage of hMDMs that produced the indicated cytokines was determined by flow cytometry. Data represent the percentage of positive cells, and asterisks indicate statistical significance compared with control cells suspended in RPMI or DMSO (* p ≤ 0.05; ** p ≤ 0.01).

To determine if Sm and Sb have cytotoxic effects, we carried out dose-time-response curves, and the viability was measured using the neutral red assay. hMDMs were stimulated with different doses (from 50–250 μM) of Sm or Sb. As shown in Fig 1A, the viability of Mø was not affected by treatment at 50 and 100 μM. However, at higher concentrations (150–250 μM) after 24 h, both compounds showed cytotoxic effects after 6 h of incubation in comparison with unstimulated cultures. A dose-time-dependent cytotoxicity effect of treatment was more evident in Mø stimulated with Sm than in those stimulated with Sb. In Mø simulated with Sm and Sb at concentrations of 50 and 100 μM respectively, the reduction in cell viability was not significant. No cytotoxic effect was observed in the hMDM cultures treated with 1% [v/v] DMSO used to solubilize Sm and Sb.

The immunostimulatory effect of Sm or Sb treatment on the expression of proinflammatory cytokines was evaluated by flow cytometry (Fig 1B). The treatment with Sm or Sb induced the expression of NF-κB at all the used doses. However, this effect was only significant in Mø treated with 100 μM Sm (p ≤ 0.01). The response to the treatment was heterogeneous among the samples. However, in all cases, we observed induction of TNF-α, IFN-γ, and IL-12. In Mø stimulated with Sm, the expression of IL-12 was higher than that observed in Mø stimulated with Sb (p ≤ 0.05).

**Fig 2:**
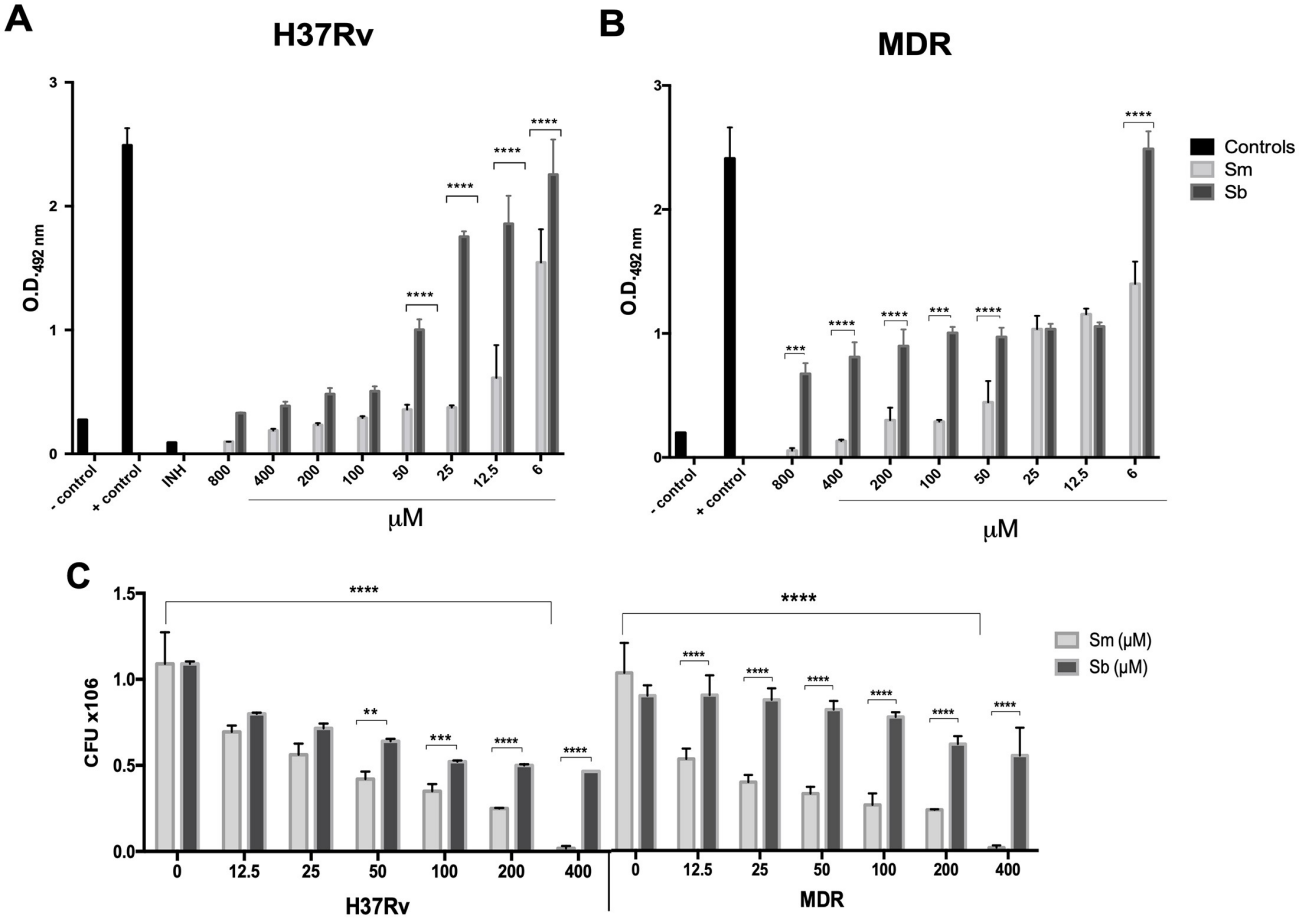
*In vitro* antimycobacterial activity of silymarin (Sm) and silibinin (Sb) tested by MIC assay and confirmed by quantification of colony forming units (CFU). The drug-sensitive strain H37Rv and the MDR strain at a concentration of 1.5 x 10^5^ bacteria per well were incubated with serial dilutions of Sm or Sb for 7 days. Medium 7H9 alone was used as a negative control (C-), bacteria without treatment were used as a positive control (C +), and 0.05 mg/L of isoniazide (INH) and 0.1% of DMSO were used to dissolve the flavonoids. (A). The upper panel shows the MIC determined as the lowest concentration of the agent that inhibits visible bacterial growth for *M*. *tuberculosis* H37Rv or MDR. (B). The lower panel shows the antimicrobial activity confirmed by CFU determination. A dose dependent effect of Sm in both the drug-susceptible H37Rv strain and the MDR strain was found. Sb showed microbicidal effect on strain H37Rv and was less effective against the MDR strain. Data represent the mean ± SEM of four independent determinations (asterisks represent a significant difference, *p* ≤ 0.05).

Next, we tested whether these flavonoids had a microbicidal effect against mycobacteria, determining their *in vitro* activity on *M*. *tuberculosis* H37Rv and the MDR strains. Bacilli were incubated with various concentrations of Sm or Sb (6–800 μM), and we found a dose dependent effect for Sm in both drug-susceptible H37Rv and MDR strains. Sb showed a microbicidal effect on strain H37Rv and was less effective against the MDR strain (Fig 2A). Regarding Sm, a significant effect at a dose above 50 μM in both strains was found. For Sb, there was a decrease of over 50% only in strain H37Rv from a concentration of 50 μM. These results were confirmed by counting colony forming units (CFUs), which confirmed the dose dependent effect of Sm in both strains and the more efficient activity of Sb against strain H37Rv. In both strains, the bactericidal effect of Sm was higher than that of Sb (Fig 2B).

**Fig 3:**
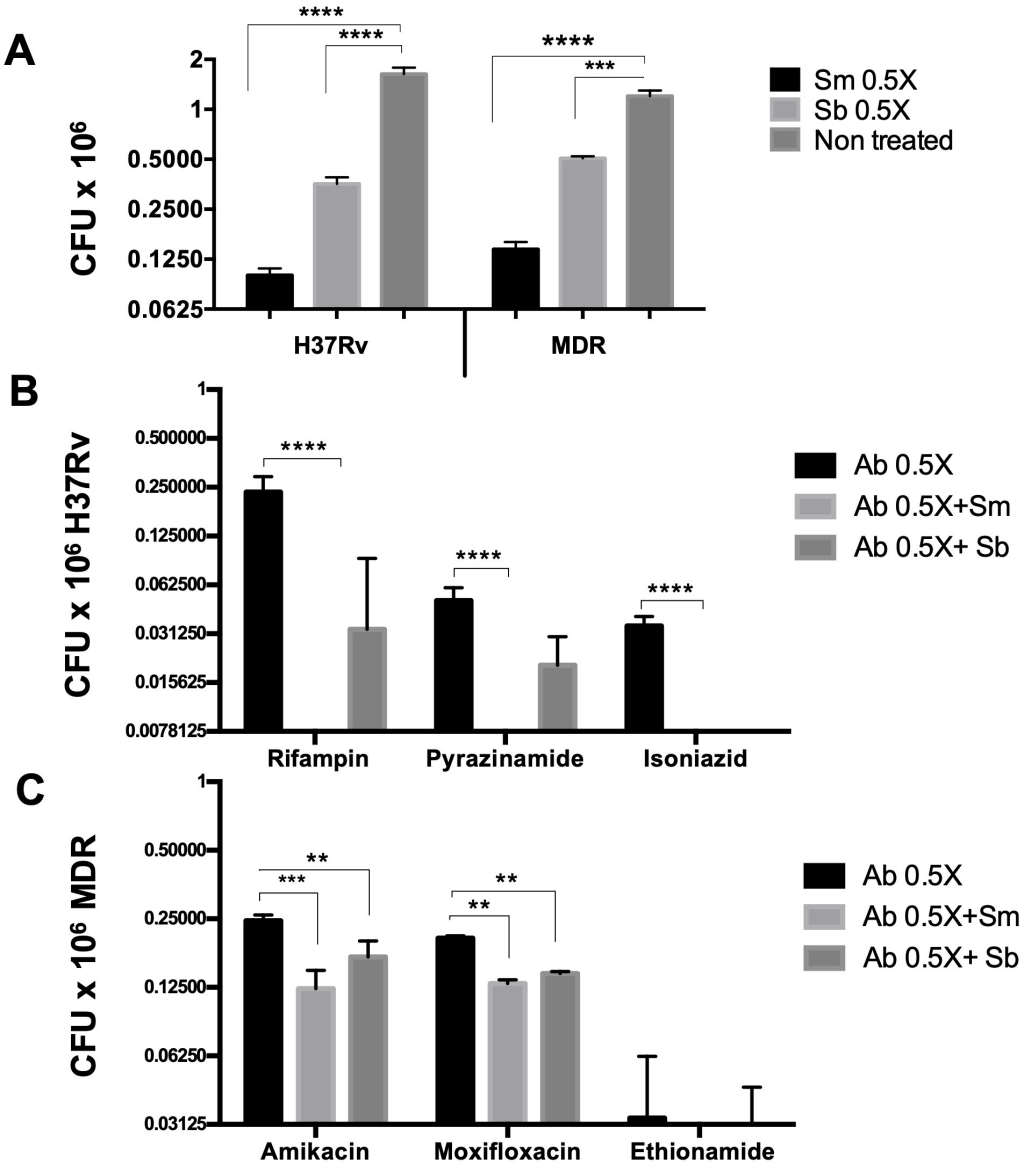
Determination *in vitro* of the synergistic activity of silymarin (Sm) and silibinin (Sb) with antituberculous drugs. The Sm and Sb antimicrobial activity was determined by CFUs (A) using the MIC determination. The viability determined by CFUs of the *M*. *tuberculosis* H37Rv (B) or MDR strain (C) was evaluated by using the half MIC concentration (0.5X) of the first- and second-line antibiotics. Dose 0.5X: rifampicin (0.05 μg/ml), pyrazinamide (2.8 μg/ml), isoniazid (0.05 μg/ml), amikacin (2 μg/ml), ethionamide (2.6 μg/ml), and moxifloxacin (2 μg/ml), respectively, in the presence or absence of 30 μg/ml of Sm or Sb (Ab 0.5X+Sm/Sb). Using half of the recommended dose (0.5X), the bacteria were eliminated more efficiently in the presence of either flavonoid, with Sm being more efficient than Sb. Data represent the mean ± SEM of four independent determinations *p* ≤ 0.05.

In addition, we evaluated *in vitro* the effect of both flavonoids with anti-TB drugs. Each drug was tested at the reported MIC (1X) and half of this dose (0.5X) to check if there was a synergistic effect. Mycobacteria were incubated with the drug alone or in combination with Sm or Sb (Fig 3). We found that Sm and Sb had a synergic effect when they were combined with first-line antibiotics. When using the MIC reported for each drug, the bacteria were totally eliminated. However, using half of the recommended dose (0.5X), the bacteria were eliminated more efficiently in the presence of either flavonoid, with Sm being more efficient than Sb. The results were statistically significant for the three used drugs (Fig 3B). Similar results were obtained with the second-line drugs. MDR bacteria were eliminated efficiently in the presence of antibiotics, but the bacterial load was lower when drugs were combined with Sm or Sb (Fig 3C). No significant differences were observed between both flavonoids for the MDR strain. These results suggest that Sm could be used as adjuvant therapy in TB treatment; however, it would be necessary to try lower doses to corroborate this effect.

**Fig 4:**
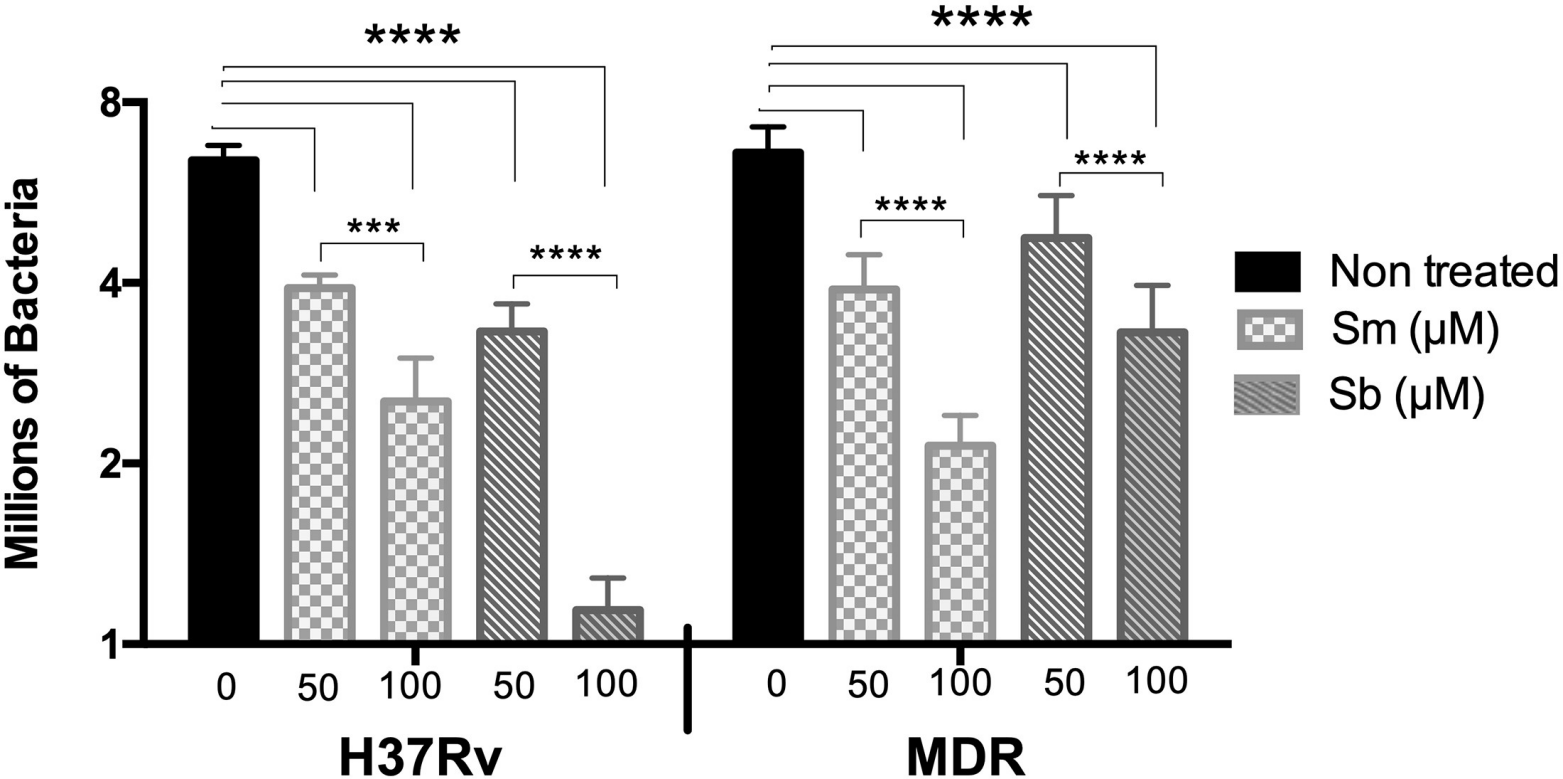
Effect of silymarin and silibinin on the bacterial burden in macrophages infected with drug-sensitive or drug-resistant mycobacteria. hMDM cultures were treated with 50 or 100 μM of silymarin or silibinin and then infected with the drug-sensitive *M*. *tuberculosis* H37Rv or the MDR strain at MOI 5:1 for 24 h. The bacterial burden was determined by CFU counts. Mø infected with drug-sensitive or drug-resistant strains and treated with Sm or Sb showed significant lower bacillary loads than untreated groups. Data represent the mean ± SEM of four independent determinations. Asterisks represent statistical significance (**** p ≤ 0.0001).

The effect of Sm or Sb on mycobacterial viability was also evaluated in infected hMDMs incubated with 50 or 100 μM, which were not toxic for macrophages. hMDMs were infected with *M*. *tuberculosis H37Rv* or the MDR clinical isolate, and the bacillary loads were determined 24 h postinfection. Mø infected with drug-sensitive or drug-resistant strains and treated with Sm or Sb showed significantly lower bacillary loads (p ≤ 0.0001) than did the untreated groups (Fig 4). In Mø infected with *M*. *tuberculosis* H37Rv, a dose-dependent response was observed. Mø treated with 100 μM Sm showed a higher reduction in bacillary loads in comparison with Mø treated with 100 μM Sb (p ≤ 0.001) and with Mø treated with 50 μM Sb (p ≤ 0.01). The effect was also dose dependent in Mø infected with the MDR strain, with the effect being higher for Sm than Sb at both doses. The lowest MDR bacterial counts were seen on Mø treated with 100 μM of Sm (Fig 4).

### *In vivo* studies

**Fig 5:**
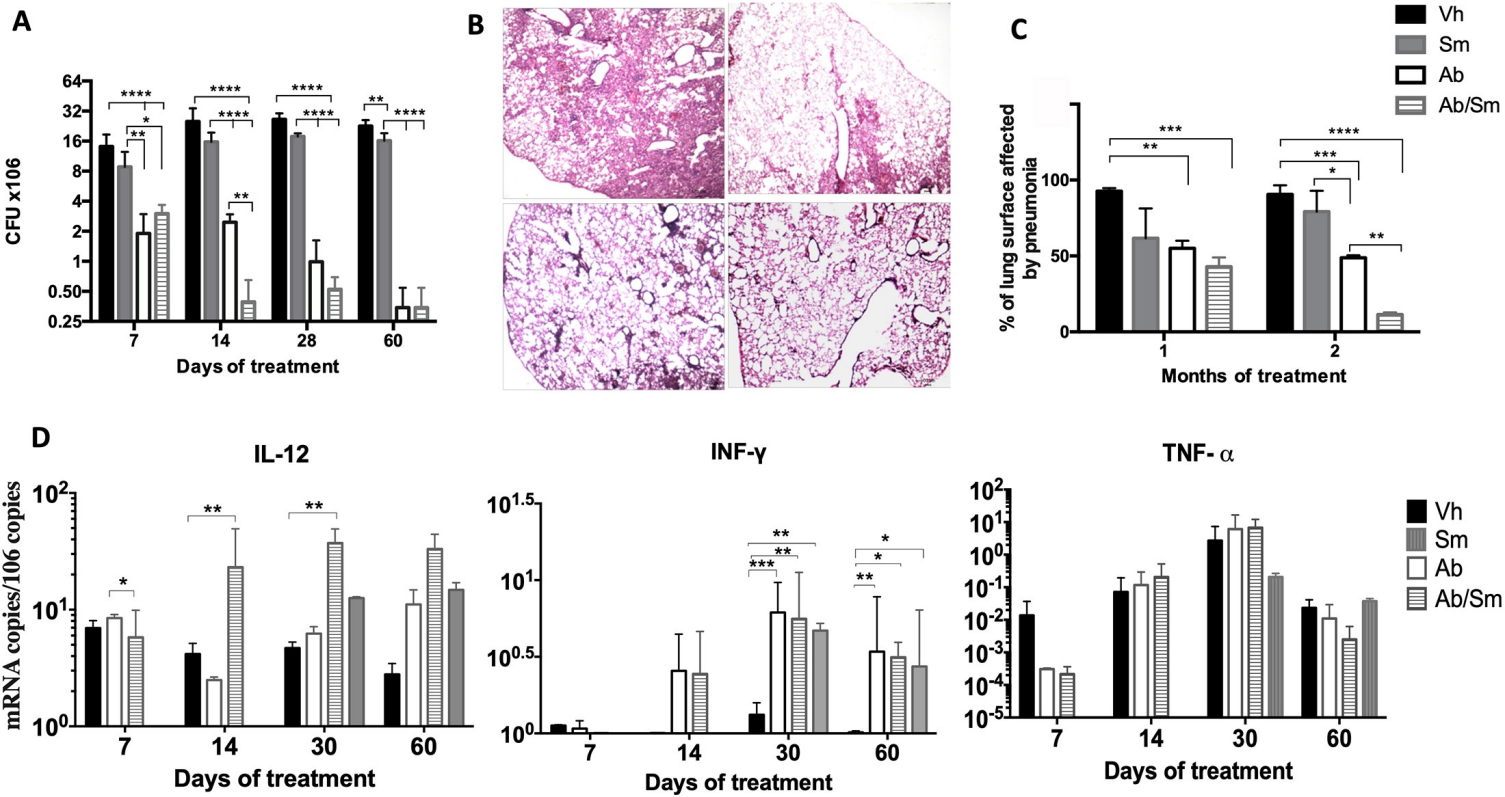
Effect of silymarin alone and as an adjunct to conventional chemotherapy in mice infected with a drug-sensitive *M*. *tuberculosis* strain. A) Groups of mice were treated after 60 days of infection with silymarin (gray bars), vehicle (control, black bars), conventional chemotherapy (white bars), or silymarin plus antibiotics (hatched bars). Mice were sacrificed on the indicated days after treatment, and right lungs (n 4 per time point/group) were used for determination of mycobacterial loads by colony forming units (CFUs). B). Representative low power micrographs at 60 days posttreatment show extensive pneumonic areas in the control group, while there are lesser pneumonic areas in the other groups, particularly in mice that received the combined treatment (all micrographs at 40x magnification, H/E staining) C). Percentage of pneumonic area determined by automated morphometry at 30 and 60 days of treatment. D). Expression of protective cytokines determined by RT-PCR. Asterisks represent statistical significance (p<0.05).

To evaluate the *in vivo* role of Sm, BALB/c mice after two months of intratracheal infection with a high dose of the *M*. *tuberculosis H37Rv* strain were treated for two months with Sm alone or in combination with antibiotics. In comparison with control mice, animals treated only with Sm showed a significantly decreased number of live bacilli in the lungs after 1 and 2 months of treatment (Fig 5A). Moreover, when mice were treated with Sm combined with antibiotics, we found a lower bacteria load than that in mice that received only the antibiotic treatment. These results were observed from the early stages of treatment; however, the difference was significant after 14 days of treatment. These findings well correlated with the morphometric analysis; there was a significant decrease in the lung area affected by pneumonia in mice treated with Sm compared with the control group (Fig 5B). The percentage of lung surface affected by pneumonia was slightly higher in mice that received Sm than in mice treated only with antibiotics. Interestingly, the percentage of the lung surface affected by pneumonia was lower in mice treated with Sm and antibiotics, suggesting an adjuvant effect of Sm (Fig 5C).

To test if Sm has an immunoregulatory effect during TB treatment, cytokine expression was determined by RT-PCR. Mice treated with antibiotics or antibiotics plus Sm showed an increase in IL-12 expression at 14 and 28 days, which was higher in animals with the combined treatment at day 14. Both groups showed higher expression of IL-12 than the controls did (Fig 5D). Similarly, mice that received the combined treatment showed a significant increase in INF-μ expression compared to that in mice treated only with antibiotics or nontreated control animals (Fig 5B). Although the TNF-μ expression was low, there was a higher expression in the group receiving combined treatment after 14 days, and after one month of treatment, the expression of this cytokine was higher in animals that received the combined treatment or antibiotics alone than in the control group. Thus, Sm has a stimulating effect on protective TB immunity *in vivo*.

**Fig 6:**
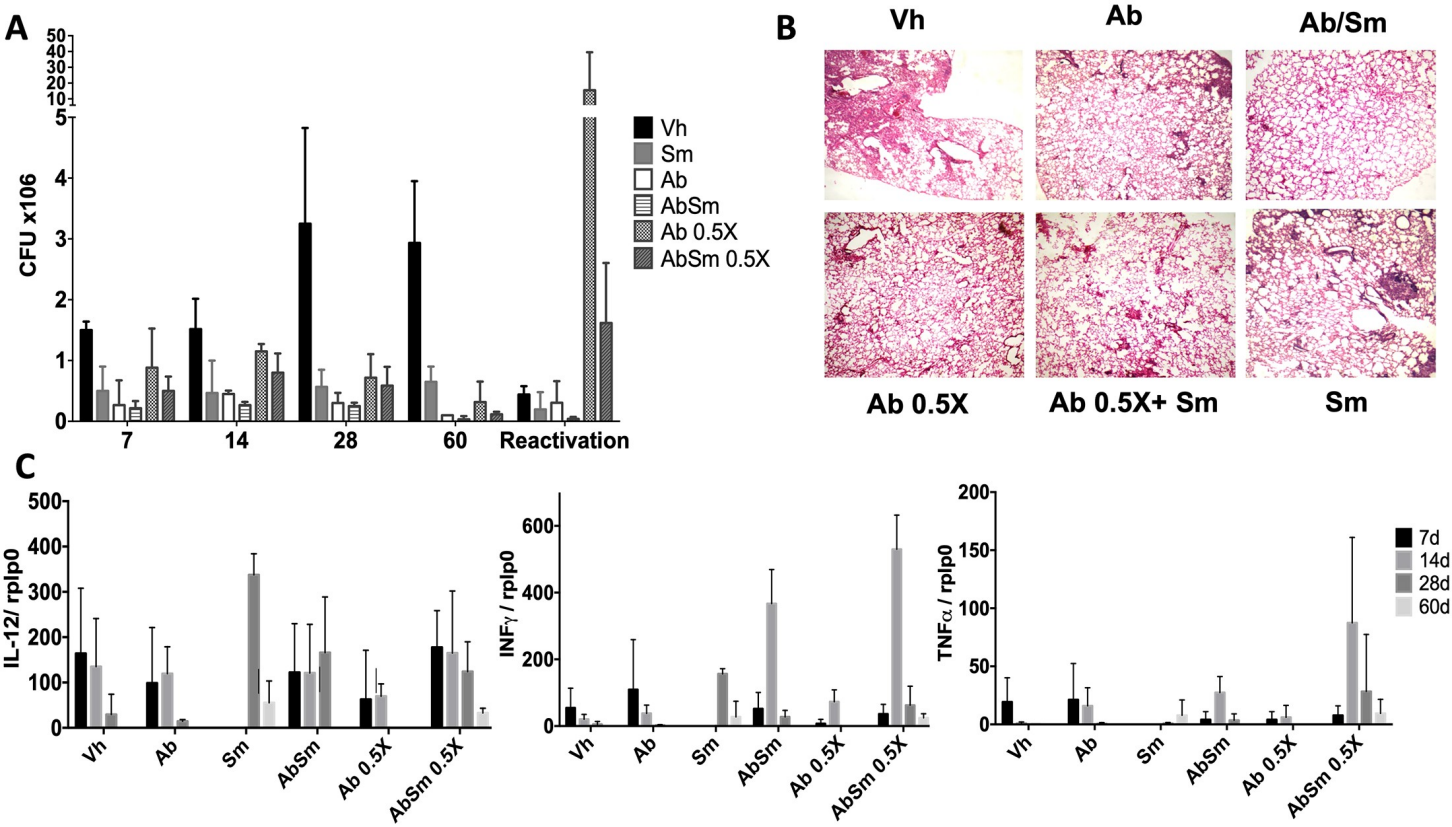
Effect of silymarin alone and as adjunct to second line antibiotics in mice infected with a drug-resistant M. tuberculosis strain. A) Groups of mice were treated after 60 days of infection with silymarin (gray bars), vehicle (control, black bars), the second line antibiotics moxifloxacin, ethionamide and pyrazinamide (white bars), silymarin plus antibiotics (hatched bars), half of the recommended doses of second line antibiotics (dotted bars) or with silymarin plus half the recommended doses of antibiotics (stippled bars). Mice were sacrificed, and lungs (n = 4 per time point/group) were used for the determination of mycobacterial loads by colony forming units (CFUs). B). Percentage of the pneumonic area determined at 30 and 60 days of treatment. Representative low power micrographs at 60 days posttreatment. C). Expression of protective cytokines determined by RT-PCR. Asterisks represent statistical significance (p<0.05).

Treatment for MDR-TB is longer (1.5–2 years) and more toxic than conventional chemotherapy because is necessary to administer 6 or more antibiotics. Thus, it is important to shorten or reduce the antibiotic doses. Following the demonstration of the good immunotherapeutic effect of Sm in animals infected with strain H37Rv, we evaluated the efficacy of Sm administration as an adjunct treatment with second line chemotherapy (moxifloxacin, ethionamide and pyrazinamide). To study this aspect, groups of mice were infected with the MDR Mtb strain (CIBIN 99) and at 60 days postinfection, some mice were given concurrent treatment with both antibiotics and Sm. The controls included mice treated with half of the recommended doses of second-line antibiotics and animals that received only the vehicle. Mice were then sacrificed at the same time points as in the previous experiments. The combined treatment with both Sm and antibiotics was the most efficacious regimen in reducing the Mtb bacterial load. Mice treated with Sm showed a significant decrease in the number of bacilli in the lungs. When using half of the recommended dose (0.5X) combined with Sm, we observed no significant differences in the bacterial loads compared with when antibiotics alone at a normal dose were administered, which suggests that Sm potentiates the effect of second-line antibiotics for the elimination of MDR strains. An additional trial was conducted to determine if TB could be reactivated after finishing treatment with Sm. To study this important point, tuberculous mice were treated for two months with Sm, and subsequently, they were left for one month without any treatment. Then, these animals received cortisone for one month to induce immune-suppression; then, they were sacrificed and their lungs used to determine live bacilli by CFU counts. There were no statistically significant changes in the number of bacteria present in the lungs when the mice were treated, except in mice treated with half the dose of antibiotics (Ab 0.5X). However, the number of bacteria in the lungs was lower when the antibiotics were combined with Sm (AbSm 0.5X) (Fig 6A). Additionally, there was a significant decrease in the lung area affected by pneumonia in mice treated with Ab+Sm (Fig 6B). Sm also induces a higher expression of protective cytokines alone or combined with Abs (Fig 6C).

## Discussion

Anti-TB drugs are, in general, an effective treatment. However, they may cause side effects [48,49]. The long-term administration of INH and RIF can induce liver injury by a high production of oxygen free radicals and leads to hepatic dysfunction [50]. Thus, it is important to obtain shorter and less-toxic therapeutic regimens [51,52] that can also be used for preventive therapy [40]. Natural products are an attractive alternative in the search for new drugs with antibiotic [16, 53], antioxidant or hepatoprotective effects to treat the rapidly growing numbers of cases of MDR and XDR TB [54]. This is the case with a standardized extract from Sm, which is safe (low side-effect profile) and has been used as antioxidant and hepatoprotective agent for liver [55, 56]. The molecular basis for the mechanism of action of Sm and Sb is not completely understood, but their hepatoprotective activity has been related to antioxidant properties, such as the induction of increased activity of superoxide dismutase in lymphocytes and the inhibition of iNOS expression [30]. This hepatoprotective effect has been demonstrated in many reports, which demonstrates that Sm induces a significant reduction in liver injury, superoxide and peroxynitrite production [26, 57, 58]. Sm could also reverse cellular membrane damage and mitochondrial damage and decrease apoptosis [59]. Sb decreases liver injury due to PZA [60]. Indeed, the coadministration of Sm with antibiotics significantly reduces liver damage in rats treated with the standard anti-TB therapy [41]. It has also been proved that Sm has some cytotoxicity and genotoxicity in different models [6, 61]. In patients, the hepatoprotective properties of Sm during TB treatment have been recently evaluated [41], with controversial results. One study demonstrated a protective effect in a small cohort of TB patients, and Sm was associated with the restoration of superoxide dismutase levels [43]; in contrast, other studies have not found any effect [21]. Perhaps, the different doses used or the coadministration of vitamin C (as an antioxidant) could mask the effect in the control group [22]. Thus, well-controlled clinical studies are needed to properly evaluate the hepato-protective effect of Sm, which has already been approved for clinical use [62].

Sm has also been shown to have immunoregulatory [30], anti-inflammatory [34] and antioxidant properties [25, 55]. Regarding to the immunoregulatory effects, Sm polarizes the immune response in a dose-dependent manner, favoring the T_H_2 immune pattern [39]. Sm induces TGF-β1 expression (which contributes to its anti-inflammatory effects) in specific cells such as mast cells, inhibiting the NF-κB pathway [11, 63], and in Kupffer cells decreases the lipoxygenase pathway affecting prostaglandin release and leukotriene synthesis [36]. All these effects are deleterious in the immune protection of TB because in humans and mice, it is well established that Th-1 and activated macrophages that actively produce NO and oxygen free radicals are essential to eliminate mycobacteria [64, 65]. In contrast, other studies have shown that silymarin induces TNF-α and inhibits IL-10 production by monocytes [66], as well as induces IFN-γ production by T-cells [67]. After the confirmation that Sm and Sb are not cytotoxic at concentrations lower than 150 μM in MDM, we observed that treatment with Sm or Sb at a concentration of 50 or 100 μM induced a significant reduction of CFUs in infected macrophages with drug-sensitive or MDR strains. Interestingly, Sm also induced the production of the protective cytokines TNFα, IL-12 and IFNγ and showed a synergistic activity *in vitro* with first- and second-line antibiotics. Other previously reported effects of Sm, such as iron chelating activity and the induction of apoptosis and autophagia [68], should also contribute to efficient mycobacterial killing by infected macrophages [66]. Thus, these results suggest that low doses of Sm or Sb *in vitro* could enhance the protective immune response against mycobacterial infection mediated by macrophages. It is important to note that the doses used in many of the studies that have reported an inhibitory effect of Sm or Sb on the production of immune suppressive cytokines are higher than 100 μM (between 100–250 μM). Thus, the cell type and concentrations should contribute to the immunoregulatory activity of Sm and Sb.

We also found a dose-dependent direct effect that decreased the viability of *Mtb in vitro*, mainly by Sm. This effect was observed using doses higher than 50 μM. Interestingly, it was more evident in an MDR strain than in the drug-sensitive strain *M*. *tuberculosis* H37Rv. The antimicrobial activity of Sm has been previously reported against *Escherichia coli*, *Bacillus subtilis* and *Staphylococcus epidermidis* [69, 70]. The mechanism of the microbicidal action of Sm has not been identified, but the presence of hydroxyl phenolic groups in flavonoids could interfere with some bacterial enzymes, such as the reported inhibitory effect on the DNA topoisomerase activity [15]. More studies are needed to determine why Sm has a greater effect than Sb alone.

Our *in vitro* studies suggest that Sm can also contribute to TB treatment as a coadjuvant agent, showing synergistic properties when combined with primary and second-line anti-TB antibiotics, as reported for the treatment of oral infections and methicillin-resistant *Staphylococcus aureus* [71]. This synergistic activity would result in enhanced killing of bacteria and prevention of drug resistance [72]. In addition, this coadjuvant activity can contribute to lowering the drug dose, and the side effects would also decrease [73]. Therapy with flavonoids combined with commonly used antibiotics is well supported in the literature, emerging as an important complementary treatment modality [69]. Regarding to the mechanism by which Sm synergizes with antibiotics, it has been reported that phenolic compounds can form complexes with proteins associated with the cell wall and alter the permeability of the cell membrane to favor the entry of antibiotics [74]

The effects of Sm against mycobacteria *in vitro* were confirmed *in vivo* by using Sm to treat BALB/c mice infected with drug-sensitive H37Rv or MDR strains. Sm produced a significant decrease in bacillary loads in the lungs and an increase in the expression of protective cytokines. In addition, we found that the combination of Sm with conventional and second-line chemotherapy had a synergistic effect, eliminating bacteria in a more efficient way than drugs alone. Adjunct therapy has been explored as a potential therapeutic strategy [75], because it may achieve better clinical outcomes in combination with standard chemotherapy [76]. These agents could shorten and improve treatment even in MDR-TB [14, 77]. Our results suggest the possibility of using Sm as an adjuvant to antibiotic therapy, even against MDR-TB. The treatments need to be inexpensive, as TB primarily affects people from developing countries [2], and the treatments should be available for health systems to reach and treat more individuals [78]. Sm is in fact an inexpensive product that has been approved for human clinical trials.

In conclusion, the potential use of a plant-derived flavonoid administered alone and in conjunction with anti-TB drugs was studied. Our results from *in vitro* and *in vivo* studies showed that Sm reduced mycobacterium viability and induced the expression of the proinflammatory cytokines TNF-α and IFN-γ, favoring a T_H_1 immune response that significantly contributed to control infections with drug-sensitive and drug-resistant mycobacteria. In addition, Sm potentiated the effect of primary and second-line antibiotics. All these effects, besides the previously reported activity in the prevention of liver damage caused by anti-TB drugs, suggest a potential use of Sm as a complementary treatment for this ancient and significant infectious disease. The major issue to solve with the use of Sm is its poor absorption and low solubility in water, which may influence its properties.

## Supporting information

S1 Table Data to determinate cell viability in noninfected human macrophages and stimulated with the indicated concentrations of silymarin (Sm) or silibinin (Sb). (PDF)

S2 Table Compilation of data to determinate in vitro antimycobacterial activity of silymarin (Sm) and silibinin (Sb) tested by MIC assay. (PDF)

S3 Table Data to determinate the synergistic activity *in vitro* of silymarin (Sm) and silibinin (Sb) with antituberculous drugs. (PDF)

S4 Table Data to determinate the effect of silymarin and silibinin on the bacterial burden in macrophages infected with drug-sensitive or drug-resistant mycobacteria. (PDF)

S5 Table Dates to evaluate the role of silymarin as an adjunct to conventional chemotherapy in mice infected with a drug-sensitive *M*. *tuberculosis* strain. (PDF)

S6 Table Effect of silymarin alone and as adjunct to second line antibiotics in mice infected with a drug-resistant M. tuberculosis strain. (PS)
