# Supplementary material for: A significant therapeutic effect of silymarin administered alone, or in combination with chemotherapy, in experimental pulmonary tuberculosis caused by drug-sensitive or drug-resistant strains: In vitro and in vivo studies
Source: PLoS One. 2019 May 30;14(5):e0217457. doi: 10.1371/journal.pone.0217457 (PMC6542514; doi:10.1371/journal.pone.0217457)
Supplement: S1 Table — (PDF) [file pone.0217457.s001.pdf]

S1 Table. Data to determinate cell viability in noninfected human macrophages and stimulated with the indicated concentrations of silymarin (Sm) or silibinin (Sb)

	Sm																		Average of the% for Sm µM					
Time	Non treated			50 µM			100 µM			150 µM			200 µM			250 µM			**0**	**50**	**100**	**150**	**200**	**250**
0	100	100	100	88	102	94	77	84	80	73	82	80	72	77	77	64	67	67	100	94.9	80.3	78.3	75.2	66.1
3	100	100	100	98	89	96	79	74	76	73	73	72	69	58	63	56	48	55	100	94.2	76.2	72.8	63.6	53.1
6	100	100	100	77	118	93	64	101	82	61	88	70	47	73	59	44	70	56	100	95.8	82.6	72.9	59.8	56.7
12	100	100	100	87	103	94	64	80	80	62	71	64	47	56	52	36	41	38	100	94.7	74.6	65.5	51.4	38.3
24	100	100	100	98	90	101	79	73	79	64	57	65	21	18	21	19	17	18	100	96.2	77.2	62.2	20.1	18
48	100	100	100	89	90	91	69	69	69	36	38	37	23	22	22	12	14	13	100	90.1	69.1	37.1	22.5	13.1

	Sb																		Average of the % for Sb µM					
Time	Non treated			50 µM			100 µM			150 µM			200 µM			250 µM			**0**	**50**	**100**	**150**	**200**	**250**
0	100	100	100	105	86	95	98	83	88	89	82	89	69	76	73	70	78	74	100	95.4	89.8	86.7	72.6	74.1
3	100	100	100	91	89	90	91	95	79	83	84	81	50	90	68	62	63	62	100	89.9	88.5	82.5	69.4	62.4
6	100	100	100	88	85	96	87	79	85	85	75	78	55	53	58	53	49	57	100	90	83.6	79.3	55.2	52.7
12	100	100	100	91	82	98	81	72	92	82	70	81	62	56	71	54	50	62	100	90.4	81.9	77.8	63	55.3
24	100	100	100	88	84	87	81	76	79	73	69	70	65	66	65	25	27	27	100	86.3	78.8	70.5	65.1	26.3
48	100	100	100	84	80	91	72	71	80	69	68	75	49	48	55	23	17	24	100	85.1	74.2	70.6	50.6	21.1

Data to determinate production of cytokines in noninfected human macrophages and stimulated with the indicated concentrations of silymarin (Sm) or silibinin (Sb)

	NF-kB						IFN-γ						TNFα						IL-12					
	RPMI	DMSO	SM 50	SM 100	SB 50	SB 100	RPMI	DMSO	SM 50	SM 100	SB 50	SB 100	RPMI	DMSO	SM 50	SM 100	SB 50	SB 100	RPMI	DMSO	SM 50	SM 100	SB 50	SB 100
S1	2.2	7.6	20	12	15	17	1.1	4	6.9	21	11	22	1	3.4	6.2	21	13	26	3.1	6.4	16	16	19	21
S2	2.4	4.9	40	53	26	8.3	6.2	9.7	47	41	47	16	7	11	59	59	57	18	3.1	5.5	28	23	5.4	19
S3	8.1	7.7	13	37	27	31	10	16	24	31	37	24	14	20	26	45	46	25	8.5	11	44	34	6.8	21
S4	4.4	11	25	21	24	29	9.6	4.4	73	64	62	60	10	9.4	24	29	45	51	3.7	14	22	15	29	11
