# Supplementary material for: A significant therapeutic effect of silymarin administered alone, or in combination with chemotherapy, in experimental pulmonary tuberculosis caused by drug-sensitive or drug-resistant strains: In vitro and in vivo studies
Source: PLoS One. 2019 May 30;14(5):e0217457. doi: 10.1371/journal.pone.0217457 (PMC6542514; doi:10.1371/journal.pone.0217457)
Supplement: S2 Table — (PDF) [file pone.0217457.s002.pdf]

S2 Table. Data to determinate in vitro antimycobacterial activity of silymarin (Sm) and silibinin (Sb) tested by MIC assay and confirmed by quantification of colony forming units (CFU).

H37Rv														
- C	0.288	0.283	0.255	0.259	0.29	0.3	0.2792							
+ C	2.565	2.374	2.367	2.683	2.57	2.26	2.4693							
INH	0.093	0.195	0.163	0.173	0.13	0.2	0.1597							
	Sm treated (OD)						Sb treated (OD)						Sm	Sb
800	0.097	0.137	0.0795	0.102	0.13	0.12	0.314	0.321	0.332	0.304	0.341	0.317	0.11	0.322
400	0.172	0.164	0.1959	0.191	0.18	0.2	0.413	0.342	0.293	0.419	0.418	0.509	0.184	0.399
200	0.213	0.192	0.243	0.219	0.29	0.2	0.461	0.488	0.421	0.546	0.526	0.502	0.226	0.491
100	0.315	0.317	0.29	0.305	0.29	0.27	0.424	0.51	0.51	0.469	0.529	0.507	0.297	0.492
50	0.342	0.36	0.331	0.386	0.4	0.31	0.959	0.903	0.969	1.113	1.052	1.124	0.355	1.02
25	0.362	0.353	0.344	0.386	0.4	0.4	1.644	1.7226	1.807	1.64	1.84	1.781	0.374	1.739
12.5	0.677	0.452	0.569	0.62	0.78	0.69	1.683	1.652	1.663	1.732	2.2	2.022	0.632	1.825
6	1.682	1.493	1.582	1.36	1.79	1.73	2.466	2.226	2.682	2.366	2.016	2.182	1.606	2.323

MDR														
- C	0.185	0.193	0.192	0.199	0.21	0.206								
+ C	2.413	2.26	2.472	2.876	2.57	2.062								
	Sm treated (OD)						Sb treated (OD)						Sm	Sb
800	0.071	0.045	0.059	0.063	0.08	0.043	0.71	0.771	0.594	0.754	0.683	0.605	0.059	0.686
400	0.127	0.132	0.151	0.159	0.14	0.134	0.897	0.771	0.625	0.867	0.892	0.855	0.141	0.818
200	0.235	0.211	0.208	0.399	0.36	0.397	1.13	0.804	0.876	0.895	0.817	0.891	0.302	0.902
100	0.305	0.297	0.302	0.268	0.29	0.313	0.963	1.068	0.953	0.981	1.051	1.017	0.296	1.006
50	0.318	0.456	0.327	0.591	0.45	0.551	0.946	0.908	1.006	1.013	1.101	0.923	0.448	0.983
25	1.031	0.987	1.004	1.116	1.16	0.908	1.106	1.135	1.131	1.172	1.013	1.039	1.034	1.099
12.5	1.091	1.123	1.12	1.106	1.24	1.22	1.062	1.086	1.079	1.024	1.041	1.143	1.15	1.073
6	1.484	1.153	1.242	1.618	1.47	1.413	2.39	2.24	2.41	2.56	2.49	2.69	1.397	2.463

H37Rv CFU determination

	Sm (μM)		Mean	SD	Sb (μM)		Mean	SD
0	1.22	0.96	1.09	0.1838	1.22	0.96	1.09	0.18385
12.5	0.68	0.71	0.695	0.0212	0.8	0.81	0.805	0.00707
25	0.54	0.59	0.565	0.0354	0.7	0.74	0.72	0.02828
50	0.44	0.38	0.41	0.0424	0.63	0.65	0.64	0.01414
100	0.38	0.32	0.35	0.0424	0.51	0.53	0.52	0.01414
200	0.24	0.25	0.245	0.0071	0.49	0.5	0.495	0.00707
400	0.018	0.017	0.0175	0.0007	0.47	0.46	0.465	0.00707

MDR CFU determination

	Sm (μM)		Mean	SD	Sb (μM)		Mean	SD
0	0.98	1.21	1.095	0.1626	0.92	1.01	0.965	0.06364
12.5	0.52	0.61	0.565	0.0636	0.99	0.91	0.95	0.05657
25	0.38	0.45	0.415	0.0495	0.96	0.9	0.93	0.04243
50	0.32	0.38	0.35	0.0424	0.83	0.89	0.86	0.04243
100	0.24	0.32	0.28	0.0566	0.85	0.79	0.82	0.04243
200	0.24	0.26	0.25	0.0141	0.68	0.59	0.635	0.06364
400	0.014	0.021	0.0175	0.0049	0.5	0.67	0.585	0.12021
