# Supplementary material for: A significant therapeutic effect of silymarin administered alone, or in combination with chemotherapy, in experimental pulmonary tuberculosis caused by drug-sensitive or drug-resistant strains: In vitro and in vivo studies
Source: PLoS One. 2019 May 30;14(5):e0217457. doi: 10.1371/journal.pone.0217457 (PMC6542514; doi:10.1371/journal.pone.0217457)
Supplement: S3 Table — (PDF) [file pone.0217457.s003.pdf]

S3 Table. Data to determinate the synergistic activity *in vitro* of silymarin (Sm) and silibinin (Sb) with antituberculous drugs.

	Sm 0.5X			Sb 0.5X			Non treated		
**H37Rv**	0.09	0.11	0.1	0.39	0.32	0.355	1.5	1.6	1.8
**MDR**	0.13	0.14	0.16	0.49	0.52	0.51	1.1	1.3	1.2

**H37Rv**	**Ab 0.5X**			**Ab 0.5X+Sm**			**Ab 0.5X+ Sb**		
Rifampin	0.2	0.3	0.2	0	0	0	0.1	0	0
Pyrazinamide	0.06	0.04	0.05	0	0	0	0.01	0.03	0.02
Isoniazid	0.04	0.03	0.035	0	0	0	0	0	0

**MDR**	**Ab 0.5X**			**Ab 0.5X+Sm**			**Ab 0.5X+ Sb**		
Amikacin	0.23	0.26	0.245	0.15	0.1	0.12	0.2	0.14	0.17
Moxifloxacin	0.2	0.21	0.207	0.125	0.135	0.13	0.145	0.14	0.146
Ethionamide	0.05	0	0.05	0	0	0	0	0	0.05
