# Supplementary material for: A significant therapeutic effect of silymarin administered alone, or in combination with chemotherapy, in experimental pulmonary tuberculosis caused by drug-sensitive or drug-resistant strains: In vitro and in vivo studies
Source: PLoS One. 2019 May 30;14(5):e0217457. doi: 10.1371/journal.pone.0217457 (PMC6542514; doi:10.1371/journal.pone.0217457)
Supplement: S4 Table — (PDF) [file pone.0217457.s004.pdf]

S4 Table. Data to determinate the effect of silymarin and silibinin on the bacterial burden in macrophages infected with drug-sensitive or drug-resistant mycobacteria.

	H37Rv						MDR					
Control	5.8	7	6.5	6.3	6.4	6.4	7.5	7.2	6.2	6.5	5.6	6.6
Sm 50 µM	4	4	3.6	3.8	4.2	3.92	4.8	3.1	3.7	3.8	4.1	3.9
Sm 100 µM	2.1	2	2.5	2.9	3.2	2.54	2.1	2.1	1.7	2.3	2.5	2.14
Sb 50 µM	3.8	3	3.4	3.6	2.8	3.32	5.4	5.1	5.25	4.9	3.1	4.75
Sb 100 µM	1.4	1.1	1	1.2	1	1.14	3.8	3.8	3.15	3.7	2.1	3.31
