# Supplementary material for: A significant therapeutic effect of silymarin administered alone, or in combination with chemotherapy, in experimental pulmonary tuberculosis caused by drug-sensitive or drug-resistant strains: In vitro and in vivo studies
Source: PLoS One. 2019 May 30;14(5):e0217457. doi: 10.1371/journal.pone.0217457 (PMC6542514; doi:10.1371/journal.pone.0217457)
Supplement: S5 Table — (PDF) [file pone.0217457.s005.pdf]

S5 Table. Dates to evaluate the role of silymarin as an adjunct to conventional chemotherapy in mice infected with a drug-sensitive *M. tuberculosis* strain

	CFU counts																															
Day	Vh								Sm								Ab								Ab + Sm							
7	13	22	11	12	11	12	11	21	10	17	6	10	6	10	6.1	6	3	2	1	1	1	2	4	2	2	2	4	3	4	3	3	3
14	13	23	36	28	27	38	16	21	13	11	20	18	14	12	17	21	2	2	2	3	2	2	3	3	1	1	0	0	0	0	0	0
28	24	25	30	26	31	29	28	19	18	17	20	18	19	18	16	17	1	1	0	0	2	1	2	1	0	0	1	1	1	1	0	0
60	21	21	20	19	29	23	26	22	17	19	12	16	17	12	15	21	0	0	0	0	1	1	0	0	0	0	0	0	1	1	0	0

Cytokine mRNA expression/ 10^6 rplpo mRNA copies												
IL-12												
Day	Vh			Ab			Ab/Sm			Sm		
7	6	8	7	8	9	9	9	3	6	0	0	0
14	5	3	4	3	2	3	5	42	23	0	0	0
30	4	5	5	7	6	6	29	46	37	12	13	13
60	2	3	3	8	14	11	25	41	33	16	13	15
IFN-γ												
7	0	0	0	0	0	0	0	0	0	0	0	0
14	0	0	0	1	0	0	0	0	1	0	0	0
30	0	0	0	1	1	1	0	1	1	1	1	1
60	0	0	0	0	1	1	1	1	0	1	1	0
TNFα												
7	0	0	0	0	0	0	0	0	0	0	0	0
14	0	0	0	0	0	0	0	0	1	0	0	0
30	8	0	0	0	18	0	5	2	13	0	0	0
60	0	0	0	0	0	0	0	0	0	0	0	0

% Pneumonia												
Day	Vh			Sm			Ab			Ab/Sm		
30	95	91	92	64	80	41	50	60	54	42	49	37
60	95	92	84	86	63	88	50	49	47	11	11	13
